# A Phase 2, Multi-Center, Randomized, Double-Blind, Parallel-Group Trial to Evaluate the Efficacy and Safety of CKD-495 in Patients With Acute and Chronic Gastritis

**DOI:** 10.1155/cjgh/2702089

**Published:** 2025-04-10

**Authors:** Su Hyun Park, Oh Young Lee, Yong Chan Lee, Kyung Sik Park, Jong Jae Park, Moo In Park, Geun Am Song, Dong Ho Lee, Hyunsoo Jung, Sung Kook Kim, Tae Nyeun Kim, Suck-Chei Choi, Sam Ryong Jee, Jong Sun Rew, Soo Teik Lee, Eun Kwang Choi, Gwang Ho Baik, Shin Jung Park

**Affiliations:** ^1^Department of Internal Medicine, Hanyang University College of Medicine, Seoul, Republic of Korea; ^2^Department of Internal Medicine, Severance Hospital, Yonsei University College of Medicine, Seoul, Republic of Korea; ^3^Department of Internal Medicine, Keimyung University College of Medicine, Daegu, Republic of Korea; ^4^Department of Internal Medicine, Korea University Guro Hospital, Seoul, Republic of Korea; ^5^Department of Internal Medicine, Kosin University College of Medicine, Busan, Republic of Korea; ^6^Department of Internal Medicine, Pusan National University School of Medicine, Busan, Republic of Korea; ^7^Department of Internal Medicine, Seoul National University Bundang Hospital, Seongnam-si, Republic of Korea; ^8^Department of Internal Medicine, Seoul National University Hospital, Seoul, Republic of Korea; ^9^Department of Internal Medicine, Kyungpook National University School of Medicine, Daegu, Republic of Korea; ^10^Department of Internal Medicine, Yeungnam University College of Medicine, Daegu, Republic of Korea; ^11^Department of Internal Medicine, Wonkwang University College of Medicine, Iksan, Jeollabuk-do, Republic of Korea; ^12^Department of Internal Medicine, Inje University Busan Paik Hospital, Inje University College of Medicine, Busan, Republic of Korea; ^13^Department of Internal Medicine, Chonnam National University Medical School, Chonnam National University Hospital, Gwangju, Republic of Korea; ^14^Department of Internal Medicine, Chonbuk National University Hospital, Jeonju, Republic of Korea; ^15^Department of Internal Medicine, Jeju National University School of Medicine, Jeju, Republic of Korea; ^16^Department of Internal Medicine, Chuncheon Sacred Heart Hospital, Hallym University, Chuncheon, Republic of Korea; ^17^Chong Kun Dang Research Institute, Chong Kun Dang Pharmaceutical Corporation, Seoul, Republic of Korea

## Abstract

CKD-495 is a newly developed drug extracted from Cinnamomum cassia Presl. This phase II study assessed the clinical benefits of CKD-495 in the treatment of acute and chronic gastritis. This study randomly assigned 250 patients with endoscopically-proven gastric mucosal erosion to five groups. The groups received either 75 mg or 150 mg of CKD-495, 100 mg of rebamipide, 60 mg of Artemisiae argyi folium 95% ethanol ext. (20 ⟶ 1) (Stillen; Dong-A ST Co., Ltd., Seoul, Korea), or placebo for 2 weeks, respectively. The primary endpoint was the erosion improvement rate, and the secondary endpoints were erosion cure rates, improvement rates of gastrointestinal symptoms, edema, redness, and hemorrhage. Drug-related adverse events were evaluated. The endoscopic erosion improvement rate was significantly higher in the 75 mg CKD-495 group than in the other groups in both the full analysis set (73% vs. 41%, 45%, 52%, 48% for the 75 mg CKD-495, 150 mg CKD-495, placebo, 60 mg Stillen, and 100 mg rebamipide groups, respectively) and the per-protocol set (PPS) (75% vs. 37%, 45%, 51%, 50%). The cure rate of gastric erosion was significantly higher in the 75 mg CKD-495 group than in the other groups. The improvement rates of hemorrhage erosion were significantly higher in the 150-mg CKD-495 group. No significant differences were observed in the safety profiles. No serious adverse events or drug reactions were observed. These results demonstrate that 75 mg of CKD-495 has excellent efficacy for the treatment of endoscopic and symptomatic improvements for acute and chronic gastritis.

**Trial Registration:** ClinicalTrials.gov identifier: NCT03437785

## 1. Introduction

Gastritis is one of the most common diseases in Korea, and it is a heterogeneous pathological condition. Gastritis is defined as the inflammation of the gastric mucosa. Gastritis manifests with various endoscopic findings such as erosion, edema, redness, and hemorrhage. Acute exacerbation of gastritis causes distinct mucosal defects, such as erosion [1]. Inflammation of the gastric mucosa is caused by disturbances in gastric acidity and the mucus-bicarbonate barrier, which is the result of an imbalance between mucosal defensive and aggressive factors [2, 3]. Gastric mucosal injury may occur when the mucosal defensive mechanism is impaired and causes gastritis or gastric ulcer [4]. Treatment of gastritis can be classified as an inhibitor of aggressive factors and enhancement of mucosal defensive agents. Gastritis treatment is mainly based on symptom control and is similar to other gastrointestinal disorders (e.g., non-ulcer dyspepsia where administering acid-suppressing agents, modulating gastrointestinal (GI) motility, and protecting the gastric mucosa are the main treatment modalities [5].

Gastric mucoprotective agents are used in combination with acid-suppressing agents such as proton pump inhibitor(PPI) or histamine-2 receptor antarognist (H2RA) or alone. Rebamipide is a well-documented mucoprotective agent and is commonly used for the treatment of acute and chronic gastritis [6, 7]. It has been shown to lead to considerable improvement in endoscopic and histological parameters as well as in symptom relief in patients with chronic gastritis [6]. Mucoprotective agents function by facilitating the healing of gastric mucosa injury by promoting the synthesis of prostaglandin and mucous glycoproteins while also impeding the production of reactive oxygen radicals and inflammatory cytokines [8].

CKD-495 is a phytopharmaceutical derived from *Cinnamomum cassia* Presl and has been reported to have anti-inflammatory and antioxidant activities in vitro and a protective effect against gastric damage in vivo by stimulating mucus secretion [9, 10]. This new gastric mucoprotective agent mainly depends on enhancing mucosal defensive agents, while the mechanism of CKD-495 has not been fully elucidated [11].

This study aimed to demonstrate the efficacy and safety of CKD-495 compared with placebo, 100-mg rebamipide, and 60 mg Stillen for the treatment of acute and chronic gastritis by assessing the optimal dose.

## 2. Materials and Methods

### 2.1. Study Design

This study was a multicenter, randomized, double-blind, parallel-group, Phase 2 clinical trial. The study was conducted at 16 Korean hospitals from January 2018 to October 2018. The study protocol was reviewed and approved by the institutional review board of each hospital as per the ethical principles of the Declaration of Helsinki and Good Clinical Practice (Hanyang University College of Medicine (2017-04-010), Kyungpook National University School of Medicine (2017-04-012), Keimyung University College of Medicine (2017-04-013), Korea University Guro Hospital (KUGH17122), Kosin University College of Medicine (1704-005-067), Pusan National University School of Medicine (H-1704-005-067), Seoul National University Bundang Hospital (L-2017-398), Seoul National University College of Medicine (H-1704-098-847), Severance Hospital, Yonsei University College of Medicine (4-2017-0441), Yeungnam University College of Medicine (2017-04-022), Wonkwang University College of Medicine (2017-04-008), Inje University Busan Paik Hospital (17-0068), Chonnam National University Hospital (CNUH-2017-125), Jeonbuk National University Hospital (2017-04-015), Jeju National University Hospital (2017-04-009), and Chuncheon Sacred Heart Hospital (2017-41)). All participants signed an informed consent form before enrollment.

### 2.2. Study Population

The enrolled participants were male and female patients aged > 19 years who had gastrointestinal symptoms and one or more gastric erosions on baseline esophagogastroduodenoscopy (EGD) at the time of enrollment or within 7 days before enrollment. The exclusion criteria were as follows: patients who were unavailable for upper endoscopy; patients with a history of peptic ulcer or reflux esophagitis; patients who had undergone a GI surgery, such as an operation to inhibit gastric acid secretion and an esophagogastric surgery (except simple closure of peptic ulcer perforation); patients with a history of GI malignancy; Zollinger–Ellison syndrome; use of anti-thrombotic agents during the study period; patients with a history of thrombotic disorder (cerebral infarction, myocardial infarction, thrombophlebitis) or coagulation disorder; patients with abnormal alanine aminotransferase or alkaline phosphatase or serum creatinine (> 3 times the upper limits of normal levels); patients who had used any H2RAs, PPIs, antacid, prokinetics, prostaglandin analogs, or gastric mucosal protective agents within 2 weeks of the study drug administration; patients who were required to take corticosteroids, nonsteroidal anti-inflammatory drugs (NSAIDs), or aspirin during the study period; patients with known hypersensitivities to the study drug; patients with genetic disorders such as galactose intolerance, Lapp lactase deficiency, or glucose-galactose malabsorption; patients taking any other clinical trial medications within 28 days from the start date of the study; patients that were pregnant or lactating; fertile women who did not consent to use medically permitted contraceptive methods (condoms, diaphragms, oral contraceptives, injectable contraceptives, or intrauterine devices); those with any other conditions or diseases that were regarded as unsuitable by the investigator.

### 2.3. Randomization

**Figure 1:**
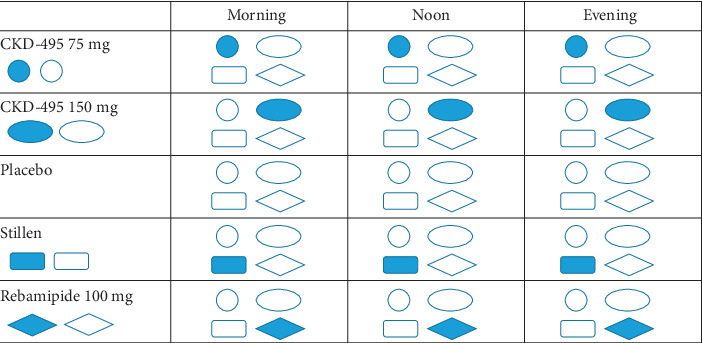
Schematic illustration of the double-blind technique in the present study.

Patients who participated in the clinical study underwent blood tests, urinalysis, and esophagogastroduodenoscopy screening tests. The eligible patients, based on the screening test results, were randomized to the test group (75 or 150 mg CKD-495) or the control group (placebo, 60 mg Stillen, and 100 mg rebamipide). A random allocation table was generated using a computer program and distributed to each institution. This study was conducted in a double-blinded manner. Participants received 75 mg CKD-495, 150 mg CKD-495, 100 mg rebamipide, 60 mg Stillen, or placebo (Figure 1). Patients visited each center for follow-up endoscopy 2 weeks after the initiation of the medications. Compliance was determined by the number of tablets remaining per drug type at the follow-up visit. Data of patients with ≥ 80% drug compliance were included in the per-protocol outcome measurements.

### 2.4. Study Assessments

#### 2.4.1. Efficacy

All patients were assessed using EGD at baseline and 2 weeks after treatment initiation. All endoscopic findings were recorded and evaluated by the principal investigators to achieve a unified result. Gastric erosion was scored from 1 to 4 (1, no visible erosion; 2, one or two erosions; 3, three to five erosions; 4, more than six erosions).

The primary endpoint was the percentage of patients with decreased erosion of more than 50%. A patient was classified as an effective case when they had a ≥ 50% reduction in their initial scores at the follow-up EGD 2 weeks after treatment initiation. The endoscopic findings after treatment were assessed as follows: effective (4–1, 3–1, 2–1, or 4–2) and ineffective (other).

The secondary efficacy endpoints were the cure rates of erosion, the improvement rate of GI symptoms, and the improvement rates of edema, redness, and hemorrhage 2 weeks after treatment initiation. (1) Cure of erosions was defined as the disappearance of all erosions. The EGD results after treatment were assessed as follows: cure (4–1, 3–1, or 2–1) and non-cure (other). (2) The improvement rate of self-reported GI symptoms was defined as a ≥ 50% reduction in the initial GI symptom scores. GI symptoms were self-reported and consisted of epigastric pain (postprandial, fasting, and nocturnal, regardless of diet), nausea/vomiting, abdominal distention, anorexia, heartburn, and belching. The frequency of each of the six GI symptoms was scored from 0 to 3 as follows: 0, absent; 1, once a week; 2, more than twice a week; 3, daily. The symptom scores were obtained from the sum of the scores, with a maximum score of 18. (3) The improvement rates of edema, redness, and hemorrhage were defined as ≥ 50% reductions in the initial scores at the follow-up EGD 2 weeks after treatment initiation. Edema was scored from 1 to 2 (1, none; 2, pale or whiter and slightly accentuated hexagonal area gastric pattern), redness from 1 to 4 (1, none; 2, minimal but obvious change; 3, conspicuous patchy discoloration; = 4, color change is beefy-red in intensity), and hemorrhage from 1 to 5 (1, none; 2, one hemorrhagic lesion; 3, 2–5 hemorrhagic lesion; 4, 6–10 hemorrhagic lesion; 5, > 10 hemorrhagic lesions or a large area of a confluent hemorrhage) [12, 13].

#### 2.4.2. Safety

Safety assessments included adverse events and adverse drug reactions, including any GI symptoms reported by the participants and abnormalities in laboratory tests or physical examinations. The safety set was composed of all participants who took the study drug at least once after randomization.

### 2.5. Sample Size and Statistical Analysis

We estimated the sample size to achieve a superiority margin, assuming that the efficacy rate of CKD-495 and placebo were 48% and 16%, respectively, based on a previous study [14]. To have a power of 90% and an *α* of 0.05 while also allowing for a 20% drop-out rate, a total of 250 patients were needed (50 per group). The analysis group was divided into the following three groups: the full analysis set (FAS), per-protocol set (PPS), and safety set. The FAS included all randomized patients who received at least one dose of the study drug and underwent at least one efficacy measurement after treatment with the study drug. The PPS included all randomized patients except those who violated the major protocol, had a compliance of less than 80%, and terminated the clinical trial ahead of schedule. The safety set included all patients who received the study drugs at least once. All efficacy endpoints were analyzed in the full analysis set and per-protocol set.

Data were analyzed using two-tailed tests with the significance set at *p* < 0.05 using SAS version 9.4 (SAS Institute, Cary, NC, USA). Continuous variables are presented as numbers, means, and standard deviations. After normality testing, continuous variables were analyzed using paired *t*-tests or Wilcoxon signed-rank tests for intragroup comparisons, and two-sample *t*-tests or Wilcoxon rank-sum tests for intragroup comparisons. Categorical variables were presented as numbers and proportions and were analyzed using Pearson's chi-square or Fisher's exact tests for intergroup comparisons.

## 3. Results

### 3.1. Patients

**Figure 2:**
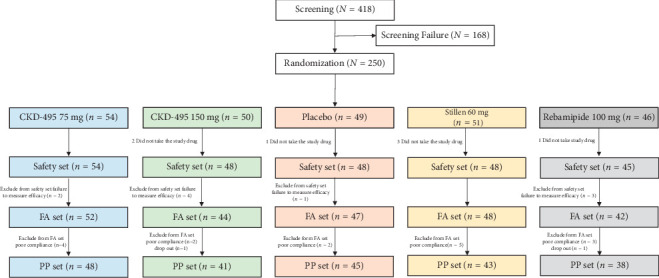
Flow chart of study population.

A total of 418 patients were screened at 16 tertiary hospitals in Korea from January 2018 to October 2018 to evaluate the efficacy and safety of CKD-495 in patients with acute and chronic gastritis. After excluding 168 patients during screening, 250 were randomly assigned to the treatment group. A total of 233 patients who received the study drug were included in the FAS. Of these, 215 were included in the PPS.  Figure 2 presents a flowchart of the study patients with detailed reasons for premature discontinuation.

### 3.2. Demographic and Clinical Characteristics

The baseline characteristics of the study population are detailed in Table 1, which shows that the study groups were comparable in terms of demographics and disease-specific characteristics. The baseline endoscopic findings (erosion, edema, redness, and hemorrhage) of the patients were well-balanced between the treatment groups (Table 2).

### 3.3. Compliance

The proportion of participants with more than 80% drug compliance throughout the treatment period was 92.3%, 95.5%, 95.75%, 89.6%, and 92.9% in the 75 mg CKD-495, 150 mg CKD-495, placebo, 60 mg Stillen, and 100 mg rebamipide groups, respectively. Drug compliance did not differ significantly between groups.

### 3.4. Primary Efficacy Assessment

**Figure 3:**
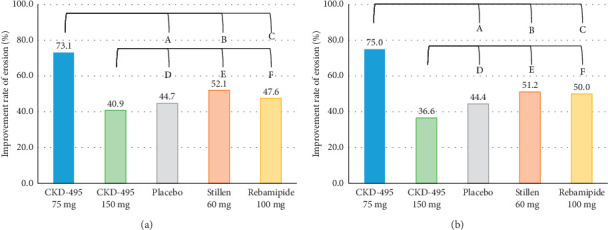
Primary endpoint; the percentage of patients with decreased erosion of more than 50%. (a) FAS. A: CKD-495 75 mg vs. placebo (*p*=0.0040), B: CKD-495 75 mg vs. Stillen 60 mg (*p*=0.0298), C: CKD-495 75 mg vs. Rebamipide 100 mg (*p*=0.0116), D: CKD-495 150 mg vs. Placebo (*p*=0.7164), E: CKD-495 150 mg vs. Stillen 60 mg (*p*=0.2832), F: CKD-495 150 mg vs. Rebamipide 100 mg (*p*=0.5311). (b) PPS. A: CKD-495 75 mg vs. placebo (*p*=0.0026), B: CKD-495 75 mg vs. Stillen 60 mg (*p*=0.0182), C: CKD-495 75 mg vs. Rebamipide 100 mg (*p*=0.0165), D: CKD-495 150 mg vs. Placebo (*p*=0.4587), E: CKD-495 150 mg vs. Stillen 60 mg (*p*=0.1786), F: CKD-495 150 mg vs. Rebamipide 100 mg (*p*=0.2289).

The improvement rate of erosion was considered the primary outcome for evaluating efficacy. The FAS included 233 patients (52 in the 75 mg CKD-495 group, 44 in the 150 mg CKD-495 group, 47 in the placebo group, 48 in the 60 mg Stillen group, and 42 in the 100 mg rebamipide group). The endoscopic erosion improvement rates in the 75 mg CKD-495, 150 mg placebo, 60 mg Stillen, and 100 mg rebamipide groups were 73%, 41%, 45%, 52%, and 48%, respectively (Figure 3(a)). In the PPS analysis, the erosion improvement rates two weeks after treatment initiation were 75%, 36.59%, 44.44%, 51.16%, and 50% in the 75-mg CKD-495, 150 mg, placebo, 60 mg Stillen, and 100 mg rebamipide groups, respectively (Figure 3(b)). A significant difference was found between the 75 mg CKD-495 and control groups in both the FAS (*p* < 0.05) and PPS (*p* < 0.05). However, no difference was found between the 150-mg CKD-495 and control groups in either the FAS or PPS.

### 3.5. Secondary Efficacy Assessment

**Figure 4:**
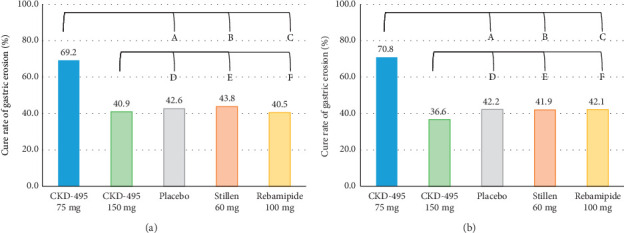
Secondary endpoint; cure rate of erosion. (a) FAS. A: CKD-495 75 mg vs placebo (*p*=0.0075), B: CKD-495 75 mg vs. Stillen 60 mg (*p*=0.0101), C: CKD-495 75 mg vs Rebamipide 100 mg (*p*=0.0052), D: CKD-495 150 mg vs. Placebo (*p*=0.8737), E: CKD-495 150 mg vs. Stillen 60 mg (*p*=0.7830), F: CKD-495 150 mg vs. Rebamipide 100 mg (*p*=0.9674). (b) PPS, A: CKD-495 75 mg vs. placebo (*p*=0.0054), B: CKD-495 75 mg vs. Stillen 60 mg (*p*=0.0053), C: CKD-495 75 mg vs. Rebamipide 100 mg (*p*=0.0073), D: CKD-495 150 mg vs. Placebo (*p*=0.5933), E: CKD-495 150 mg vs. Stillen 60 mg (*p*=0.6207), F: CKD-495 150 mg vs. Rebamipide 100 mg (*p*=0.6156).

**Figure 5:**
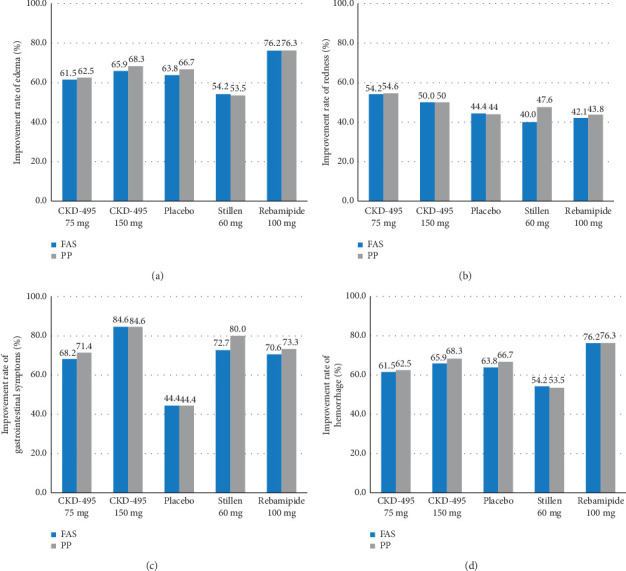
Secondary endpoint (a) Improvement rate of edema (b) Improvement rate of redness (c) Improvement rate of hemorrhage (d) Improvement rate of gastrointestinal symptoms.

The cure rate of gastric erosion was significantly higher in the 75 mg CKD-495 group than in the other groups in both the FAS (69% and 41% vs. 43%, 44%, and 41% for the 75 mg CKD-495, 150 mg CKD-495, placebo, 60-mg Stillen, and 100 mg rebamipide groups, respectively) and the PPS (71% and 37% vs. 42%, 42%, and 42%) (Figure 4). The improvement rates of hemorrhage erosion were significantly higher in the 150 mg CKD-495 group (*p*=0.0235). There were no statistically significant differences in the improvement rates of edema, redness, or gastrointestinal symptoms (Figure 5).

### 3.6. Safety Analysis

No serious adverse events were reported in any of the groups during the study period. In the safety set (*n* = 243), 23 cases of adverse events were reported in 19 patients (7.82%), and 20 cases of adverse drug reactions were reported in 16 patients (6.58%). All adverse events were mild or moderate, with no significant intergroup differences. Adverse events that did not occur in the control group and only occurred in the clinical trial groups (75 mg CKD-495 or 150 mg CKD-495) included erosive duodenitis (75 mg CKD-495), chest discomfort (75 mg CKD-495 and 150 mg CKD-495), and dizziness (75 mg CKD-495 and 150 mg CKD-495), which are all mild adverse events. No significant differences were observed among all groups in terms of vital signs (blood pressure, pulse, and body temperature), physical examination results, or laboratory test results after the administration of the clinical trial drugs (Table 3).

## 4. Discussion

This Phase 2, multicenter, randomized, double-blind, parallel-group study aimed to evaluate the efficacy and safety of CKD-495 in patients with acute and chronic gastritis. We demonstrated that 75 mg CKD-495 showed better improvement rates and cure rates of erosion in endoscopic findings than the other drugs in the control groups. The 75 mg CKD-495 was confirmed to be effective in improving erosion rates. However, 150 mg CKD-495 showed no significant differences from the control group, except for an improved rate of hemorrhage. All adverse reactions reported in the 150 and 75 mg CKD-495 groups were mild or moderate, with no significant differences between the groups. In addition, CKD-495 is considered a safe drug because no unusual adverse reactions were observed at doses of 75 and 150 mg. Therefore, 75 mg of CKD-495 is considered an appropriate dose owing to its efficacy and safety for improving erosion in patients with acute and chronic gastritis.

Stillen, an ethanol extract of *Artemisia asiatica*, has been reported to have anti-inflammatory, anti-oxidative, and cytoprotective actions in various models of gastric mucosal damage [14–17] and is currently used as a treatment for gastritis [18]. Previous studies have confirmed that the main mechanism of action of Stillen in preventing gastric mucosal injury likely involves antioxidant activity by inhibiting the production of FeSO_4_-induced reactive oxygen species, which prevents H_2_O_2_-induced gastric epithelial damage and anti-inflammatory effects by inhibiting pro-inflammatory cytokines such as TNF-α [19, 20]. In addition, Stillen is effective for gastric mucosal healing in patients with erosive gastritis, as assessed by endoscopy [14]. Interestingly, it has also been reported to have anti-oxidative and anti-inflammatory effects on experimentally induced gastrointestinal, hepatic, and pancreatic lesions [21, 22]. In particular, its effect in modulating oxidative stress in gastrointestinal disorders has been highlighted, with multi-potent natural antioxidants being designed mainly through rational design and target-based drug discovery strategies [23].

*Cinnamomum cassia* Blume is a popular traditional Chinese herbal medicine that has been used as an antipyretic, anti-rheumatic, anti-tumor, anti-spasmodic, and stomachic treatment [24–26]. *Cinnamomum cassia* has anti-ulcer activity, likely by enhancing defensive factors and inhibiting the growth of *Helicobacter pylori* and urease activity [27–29]. The extracts from *Cinnamomum cassia* exhibited antioxidant activity both in vitro and in vivo [30]. Previous studies reported that the *Cinnamomum cassia* twig and its constituents (eugenol and cinnamic acid) exhibited antioxidant activity in vitro and a protective effect against gastric damage in vivo by stimulating mucus secretion [9]. A previous study on its mechanism reported that it presented anti-inflammatory activities that suppress the release of nitric oxide and prostaglandin E2 [31].

CKD-495 is a newly developed drug extracted from *Cinnamomum cassia* Presl as a candidate drug for gastritis treatment. Its potential mechanisms of action are as follows: (1) retrieval of glutathione, an antioxidant defense system; (2) increase in the mucosal blood flow, inhibition of Prostaglandin E2 synthesis, and recovery of epithelial cell injury; (3) reduction of myeloperoxidase and neutrophil infiltration index values. In addition, in ethanol-induced gastric damage and NSAID-induced gastric damage models, CKD-495 showed non-inferiority compared with conventional drugs. Based on the proposed mechanisms of action and the results from animal and non-clinical trials, Phase 2 clinical trials were performed to compare and evaluate the efficacy and safety of CKD-495 in patients with acute and chronic gastritis.

*Helicobacter pylori* infection is a high-risk factor for chronic gastritis and peptic ulcers [32]. Chen et al. reported that 94% and 98% of the patients with gastric and duodenal ulcers, respectively, had an associated *H. pylori* infection [33]. Previous studies on the anti-inflammatory effects of rebamipide according to *H. pylori* status in patients with chronic erosive gastritis suggested that no statistically significant difference was found in the effect of rebamipide on symptoms or inflammation scores, regardless of *H. pylori* infection [34]. Although the association between gastritis and *H. pylori* infection is well known, *H. pylori* infection was not considered in this study as CKD-495 was evaluated for the treatment of gastritis, not for the treatment evaluation of *H. pylori* infection. However, the possibility of a difference in treatment effects due to *H. pylori* infection cannot be excluded; therefore, this was considered a limitation of this study.

In our study, dietary patterns was not considered in the eligibility criteria for participating in the study and not restricted during the study period. It is also a limitation of our study because it can can interference in erosion.

Cinnamomum cassia can impact on blood glucose in several ways, but diabetic condition was not included as an exclusion criterion in this study. In the Phase 2 clinical trial, 15 out of 233 (6.44%) patients had a history of diabetes, and there were no blood glucose-related problems confirmed through laboratory tests or adverse events in all subjects, including these subjects. It is our limitasion that further research will require this consideration.

The primary and secondary outcomes showed dose-independent results, which, although a limitation of this study, can be explained as follows: the therapeutic effects of CKD-495 appear to be multi-targeted owing to the presence of various compound groups, which is characteristic in extracts from natural compounds [35, 36]. The pharmacologically effective substances in CKD-495 are natural compounds that cannot be specified as a single component; therefore, several main pharmaceutical components and similar structural components appear to be present. Therefore, pharmaceutical structural compounds and similar structural components can act together on a drug target (or more than one) where the relative selectivity and affinity of each substance may differ. Drug selectivity refers to selectivity for target receptors (including enzymes) and receptor structure analogs (e.g., isoforms); affinity is defined as a 50% effective drug concentration expressed in ED50 or Kd values. Therefore, when the drug concentration increases, the saturation of each receptor may increase, resulting in a completely different reaction in the circuit of the pharmacological action of these receptors. The study suggested that *Cinnamomum* extracts may affect the inhibition of metabolites, such as CYP3A4 and CYP2D [37], and the expression levels of glucose transporter-4 [38] which may explain the dose-independent results. Owing to the important features of natural compounds, their polymolecular composition promotes a multifunctional mode of action. The therapeutic effect appears to involve multiple targets. Therefore, this may be the reason for the dose-independent results of the Phase 2 clinical trials of CKD-495. Further research is needed on the adverse effects of its long-term use at high doses and to confirm dose-independent outcomes and optimal dose. Additional studies are needed to support the clinical trial results in terms of the mechanism of action, efficacy of the drug, and ingredients of the drug.

## 5. Conclusion

This study demonstrated that 75 mg of CKD-495 is effective and safe in treating patients with acute and chronic gastritis in terms of improving endoscopic findings and GI symptoms. This study suggests that 75 mg of CKD-495 has an excellent efficacy and safety profile while representing an alternative option for treating gastritis as a mucoprotective agent.

## Figures and Tables

**Table 1:** Baseline demographic characteristics of the study patients.

Variable		Total (*n* = 233)		CKD-495 75 mg (*n* = 52)		CKD-495 150 mg (*n* = 44)		Placebo (*n* = 47)		Stillen® 60 mg (*n* = 48)		Rebamipide100 mg (*n* = 42)		*p* value
		*n* (%)		*n* (%)		*n* (%)		*n* (%)		*n* (%)		*n* (%)		
Sex	Men	122 (52.7)		25 (48.1)		24 (54.6)		32 (68.1)		21 (43.8)		20 (47.6)		0.1396^a^
	Women	111 (47.6)		27 (51.9)		20 (45.5)		15 (31.9)		27 (56.3)		22 (52.4)		

Age (yr)	Mean (±SD)	48.61 (±16.4)		45.12 (±14.5)		48.43 (±16.6)		51.38 (±17.4)		47.40 (±17.3)		51.43 (±16.5)		0.2720^b^

Height (cm)		165.60 (±8.75)		165.92 (±8.79)		165.99 (±9.2)		166.96 (±8.0)		164.92 (±9.0)		164.06 (±8.8)		0.6703^a^

Weight (kg)		66.15 (±13.3)		67.51 (±12.6)		65.57 (±12.72)		68.33 (±13.11)		64.68 (±14.33)		64.29 (±13.83)		0.5334^a^

Concurrent disease	Present	104 (44.6)		22 (42.3)		19 (43.2)		21 (44.7)		20 (41.7)		22 (52.4)		0.8541^b^

Past history	Present	77 (33.1)		15 (28.9)		11 (25.0)		20 (42.6)		17 (35.4)		14 (33.3)		0.4416^b^

Concomitant medication	Present	225 (96.57)		51 (98.08)		43 (97.73)		44 (93.62)		48 (100.00)		39 (92.86)		0.2655^b^

Gastrointestinal symptom scores		4.99 (±2.9)		4.6 (±3.1)		4.75 (±2.9)		5.11 (±2.6)		5.13 (±3.1)		5.45 (±3.0)		0.6559

Classification of gastritis	Acute gastritis	72	30.9	18	34.62	11	25.00	14	29.79	13	27.08	16	38.10	0.6578^a^
	Chronic gastritis	161	69.1	34	65.38	33	75.00	33	70.21	35	72.92	26	61.90	

Duration of gastritis	Acute gastritis	1.12 (±3.08)		2.15 (±3.32)		0.38 (±1.26)		0 (±0.01)		0.86 (±1.78)		1.65 (±5.07)		0.2854^b^
	Chronic gastritis	3.76 (±5.07)		2.05 (±2.66)		3.85 (±4.5)		4.68 (±6.42)		3.81 (±5.96)		4.65 (±4.71)		0.2175^b^

*Note:* Data are presented as mean ± SD or number (%).

^a^Chi-square test for intergroup comparison.

^b^Analysis of variance for intergroup comparison.

**Table 2:** Baseline endoscopic findings in the study patients.

Variable		Total (*n* = 233)		CKD-495 75 mg (*n* = 52)		CKD-495 150 mg (*n* = 44)		Placebo (*n* = 47)		Stillen® 60 mg (*n* = 48)		Rebamipide100 mg (*n* = 42)		*p* value^a^
		*n*	%	*n*	%	*n*	%	*n*	%	*n*	%	*n*	%	
Erosion grade (Baseline)	Grade 1	—	—	—	—	—	—	—	—	—	—	—	—	0.2177
	Grade 2	126	54.08	31	59.62	29	65.91	22	46.81	23	47.92	21	50.00	
	Grade 3	63	27.04	16	30.77	9	20.45	13	27.66	16	33.33	9	21.43	
	Grade 4	44	18.88	5	9.62	6	13.64	12	25.53	9	18.75	12	28.57	

Edema grade (Baseline)	Grade 1	161	69.10	36	69.23	33	75.00	31	65.96	33	68.75	28	66.67	0.9014
	Grade 2	72	30.90	16	30.77	11	25.00	16	34.04	15	31.25	14	33.33	—

Redness grade (Baseline)	Grade 1	119	51.07	31	59.62	20	45.45	20	42.55	25	52.08	23	54.76	0.2832
	Grade 2	78	33.48	13	25.00	18	40.91	20	42.55	17	35.42	10	23.81	—
	Grade 3	29	12.45	7	13.46	5	11.36	7	14.89	5	10.42	5	11.90	—
	Grade 4	7	3.00	1	1.92	1	2.27	0	0.00	1	2.08	4	9.52	—

Hemorrhage grade (Baseline)	Grade 1	163	69.96	33	63.46	31	70.45	35	74.47	37	77.08	27	64.29	0.3221
	Grade 2	36	15.45	14	26.92	7	15.91	4	8.51	5	10.42	6	14.29	—
	Grade 3	23	9.87	2	3.85	5	11.36	4	8.51	6	12.50	6	14.29	—
	Grade 4	7	3.00	1	1.92	1	2.27	3	6.38	0	0.00	2	4.76	—
	Grade 5	4	1.72	2	3.85	0	0.00	1	2.13	0	0.00	1	2.38	—

^a^Chi-square test for intergroup comparison.

**Table 3:** Adverse events during the study.

	Total (*n* = 243)	CKD-495 75 mg (*n* = 54)	CKD-495 150 mg (*n* = 48)	Placebo (*n* = 48)	Stillen 60 mg (*n* = 48)	Rebamipide100 mg (*n* = 45)	*p* value^a^
	*n* (%) [case]	*n* (%) [case]	*n* (%) [case]	*n* (%) [case]	*n* (%) [case]	*n* (%) [case]	
Gastrointestinal disorders	12 (4.94) [16]	3 (5.56) [5]	2 (4.17) [2]	1 (2.08) [3]	2 (4.17) [2]	4 (8.89) [4]	0.6742^a^
Gastrooesophageal reflux disease	4 (1.65) [4]	—	2 (4.17) [2]	—	1 (2.08) [1]	1 (2.22) [1]	0.3779^a^
Diarrhea	3 (1.23) [4]	1 (1.85) [2]	—	1 (2.08) [1]	—	1 (2.22) [1]	0.8420^a^
Dyspepsia	3 (1.23) [3]	1 (1.85) [1]	—	—	1 (2.08) [1]	1 (2.22) [1]	0.8420^a^
Abdominal distension	2 (0.82) [2]	1 (1.85) [1]	—	1 (2.08) [1]	—	—	1.0000^a^
Erosive duodenitis	1 (0.41) [1]	1 (1.85) [1]	—	—	—	—	1.0000^a^
Eructation	1 (0.41) [1]	—	—	1 (2.08) [1]	—	—	0.7778^a^
Gastric ulcer	1 (0.41) [1]	—	—	—	—	1 (2.22) [1]	0.1852^a^
General disorders and administration site conditions	1 (0.41) [1]	—	1 (2.08) [1]	—	—	—	0.7778^a^
Chest discomfort	1 (0.41) [1]	—	1 (2.08) [1]	—	—	—	0.7778^a^
Neoplasms benign, malignant and unspecified (incl cysts and polyps)	1 (0.41) [1]	—	—	1 (2.08) [1]	—	—	0.7778^a^
Gastrointestinal submucosal tumour	1 (0.41) [1]	—	—	1 (2.08) [1]	—	—	0.7778^a^
Nervous system disorders	1 (0.41) [1]	—	1 (2.08) [1]	—	—	—	0.7778^a^
Dizziness	1 (0.41) [1]	—	1 (2.08) [1]	—	—	—	0.7778^a^
Skin and subcutaneous tissue disorders	1 (0.41) [1]	—	—	—	1 (2.08) [1]	—	0.7778^a^
Cold sweat	1 (0.41) [1]	—	—	—	1 (2.08) [1]	—	0.7778^a^
Total	16 (6.58) [20]	3 (5.56) [5]	4 (8.33) [4]	2 (4.17) [4]	3 (6.25) [3]	4 (8.89) [4]	0.8914^a^

^a^Fisher's exact test for comparison of proportions between the groups.

## Data Availability

Due to the nature of this research, participants of this study did not agree for their data to be shared publicly, so supporting data are not available.

## References

[1] Laine L., Cohen H., Sloane R., Marin-Sorensen M., Weinstein W. M. (1995). Interobserver Agreement and Predictive Value of Endoscopic Findings for *H. pylori* and Gastritis in Normal Volunteers. *Gastrointestinal Endoscopy*.

[2] Taylor I. L. (1984). Gastrointestinal Hormones in the Pathogenesis of Peptic Ulcer Disease. *Clinics in Gastroenterology*.

[3] Defize J., Meuwissen S. G. (1987). Pepsinogens: An Update of Biochemical, Physiological, and Clinical Aspects. *Journal of Pediatric Gastroenterology and Nutrition*.

[4] Laine L., Takeuchi K., Tarnawski A. (2008). Gastric Mucosal Defense and Cytoprotection: Bench to Bedside. *Gastroenterology*.

[5] Talley N. J. (2003). Update on the Role of Drug Therapy in Non-Ulcer Dyspepsia. *Reviews in Gastroenterological Disorders*.

[6] Chitapanarux T., Praisontarangkul O. A., Lertprasertsuke N. (2008). An Open-Labeled Study of Rebamipide Treatment in Chronic Gastritis Patients With Dyspeptic Symptoms Refractory to Proton Pump Inhibitors. *Digestive Diseases and Sciences*.

[7] Han X., Jiang K., Wang B., Zhou L., Chen X., Li S. (2015). Effect of Rebamipide on the Premalignant Progression of Chronic Gastritis: A Randomized Controlled Study. *Clinical Drug Investigation*.

[8] Arakawa T., Higuchi K., Fujiwara Y. (2005). 15th Anniversary of Rebamipide: Looking Ahead to the New Mechanisms and New Applications. *Digestive Diseases and Sciences*.

[9] Jung J., Lee J. H., Bae K. H., Jeong C. S. (2011). Anti-Gastric Actions of Eugenol and Cinnamic Acid Isolated From Cinnamomi Ramulus. *Yakugaku Zasshi*.

[10] Lee J., Lim S. (2021). Anti-Inflammatory, and Anti-Arthritic Effects by the Twigs of Cinnamomum cassia on Complete Freund’s Adjuvant-Induced Arthritis in Rats. *Journal of Ethnopharmacology*.

[11] Seo S. Y., Lee S. T., Kim S. K. (2023). Efficacy and Safety of CKD-495 in Acute and Chronic Gastritis: A Phase III Superiority Clinical Trial. *Medicine*.

[12] Tytgat G. N. (1991). The Sydney System: Endoscopic Division. Endoscopic Appearances in Gastritis/Duodenitis. *Journal of Gastroenterology and Hepatology*.

[13] Kim G. H., Lee H. L., Joo M. K. (2021). Efficacy and Safety of Rebamipide Versus Its New Formulation, AD-203, in Patients With Erosive Gastritis: A Randomized, Double-Blind, Active Control, Noninferiority, Multicenter, Phase 3 Study. *Gut and Liver*.

[14] Seol S.-Y., Kim M.-H., Ryu J.-S., Choi M.-G., Shin D.-W., Ahn B.-O. (2004). DA-9601 for Erosive Gastritis: Results of a Double-Blind Placebo-Controlled Phase III Clinical Trial. *World Journal of Gastroenterology*.

[15] Kim J. H., Shin C. Y., Jang S. W. (2021). Anti-Inflammatory Effects of DA-9601, an Extract of Artemisia Asiatica, on Aceclofenac-Induced Acute Enteritis. *Korean Journal of Physiology and Pharmacology*.

[16] Kim J. S., Cha K. H., Kang S. Y. (2016). In Vivo Gastric Residence and Gastroprotective Effect of Floating Gastroretentive Tablet of DA-9601, an Extract of Artemisia Asiatica, in Beagle Dogs. *Drug Design, Development and Therapy*.

[17] Lee S., Park H. H., Son H. Y. (2007). DA-9601 Inhibits Activation of the Human Mast Cell Line HMC-1 Through Inhibition of NF-Κb. *Cell Biology and Toxicology*.

[18] Lee O. Y., Kang D. H., Lee D. H. (2014). A Comparative Study of DA-9601 and Misoprostol for Prevention of NSAID-Associated Gastroduodenal Injury in Patients Undergoing Chronic NSAID Treatment. *Archives of Pharmacal Research*.

[19] Choi E. J., Oh H. M., Na B. R. (2008). Eupatilin Protects Gastric Epithelial Cells From Oxidative Damage and Down-Regulates Genes Responsible for the Cellular Oxidative Stress. *Pharmaceutical Research*.

[20] Choi S.-C., Choi E.-J., Oh H.-M. (2006). DA-9601, a Standardized Extract of Artemisia Asiatica, Blocks TNF-Alpha-Induced IL-8 and CCL20 Production by Inhibiting P38 Kinase and NF-kappaB Pathways in Human Gastric Epithelial Cells. *World Journal of Gastroenterology*.

[21] Huh K., Kwon T. H., Shin U. S. (2003). Inhibitory Effects of DA-9601 on Ethanol-Induced Gastrohemorrhagic Lesions and Gastric Xanthine Oxidase Activity in Rats. *Journal of Ethnopharmacology*.

[22] Oh T. Y., Lee J. S., Ahn B. O. (2001). Oxidative Stress Is More Important Than Acid in the Pathogenesis of Reflux Oesophagitis in Rats. *Gut*.

[23] Mousavi T., Hadizadeh N., Nikfar S., Abdollahi M. (2020). Drug Discovery Strategies for Modulating Oxidative Stress in Gastrointestinal Disorders. *Expert Opinion on Drug Discovery*.

[24] Zhang C., Fan L., Fan S. (2019). Cinnamomum cassia Presl: A Review of Its Traditional Uses, Phytochemistry, Pharmacology and Toxicology. *Molecules*.

[25] Jung B., Shin M. (1990). *Illustrated Encyclopedia of Korean Medicinal Plants*.

[26] Lee E.-J., Chung T.-W., Lee J.-H. (2018). Water-Extracted Branch of *Cinnamomum cassia* Promotes Lung Cancer Cell Apoptosis by Inhibiting Pyruvate Dehydrogenase Kinase Activity. *Journal of Pharmacological Sciences*.

[27] Akira T., Tanaka S., Tabata M. (1986). Pharmacological Studies on the Antiulcerogenic Activity of Chinese Cinnamon. *Planta Medica*.

[28] Tanaka S., Yoon Y. H., Fukui H. (1989). Antiulcerogenic Compounds Isolated From Chinese Cinnamon. *Planta Medica*.

[29] Tabak M., Armon R., Neeman I. (1999). Cinnamon Extracts’ Inhibitory Effect on *Helicobacter pylori*. *Journal of Ethnopharmacology*.

[30] Lin C. C., Wu S. J., Chang C. H., Ng L. T. (2003). Antioxidant Activity of *Cinnamomum cassia*. *Phytotherapy Research*.

[31] Park H.-J., Lee J.-S., Lee J.-D. (2005). The Anti-Inflammatory Effect of Cinnamomi Ramulus. *The Journal of Korean Medicine*.

[32] Manabe N., Matsueda K., Haruma K. (2022). Epidemiological Review of Gastroesophageal Junction Adenocarcinoma in Asian Countries. *Digestion*.

[33] Chen X., Xia C., Li Q., Jin L., Zheng L., Wu Z. (2018). Comparisons Between Bacterial Communities in Mucosa in Patients With Gastric Antrum Ulcer and a Duodenal Ulcer. *Frontiers in Cellular and Infection Microbiology*.

[34] Du Y., Li Z., Zhan X. (2008). Anti-Inflammatory Effects of Rebamipide According to *Helicobacter pylori* Status in Patients With Chronic Erosive Gastritis: A Randomized Sucralfate-Controlled Multicenter Trial in China-STARS Study. *Digestive Diseases and Sciences*.

[35] Atanasov A. G., Zotchev S. B., Dirsch V. M. (2021). Natural Products in Drug Discovery: Advances and Opportunities. *Nature Reviews Drug Discovery*.

[36] Herranz-López M., Losada-Echeberría M., Barrajón-Catalán E. (2018). The Multitarget Activity of Natural Extracts on Cancer: Synergy and Xenohormesis. *Medicines (Basel)*.

[37] Subehan T. U., Usia T., Iwata H., Kadota S., Tezuka Y. (2006). Mechanism-Based Inhibition of CYP3A4 and CYP2D6 by Indonesian Medicinal Plants. *Journal of Ethnopharmacology*.

[38] Han Y., Jung H. W., Bae H. S., Kang J. S., Park Y. K. (2013). The Extract of *Cinnamomum cassia* Twigs Inhibits Adipocyte Differentiation via Activation of the Insulin Signaling Pathway in 3T3-L1 Preadipocytes. *Pharmaceutical Biology*.

